# A Clinicopathological Analysis of Soft Tissue Sarcoma with Telangiectatic Changes

**DOI:** 10.1155/2015/740571

**Published:** 2015-12-29

**Authors:** Hiroshi Kobayashi, Keisuke Ae, Taisuke Tanizawa, Tabu Gokita, Noriko Motoi, Seiichi Matsumoto

**Affiliations:** ^1^Department of Orthopedic Surgery, Cancer Institute Hospital, Ariake 3-8-31, Koutou-ku, Tokyo 135-8550, Japan; ^2^Department of Orthopedic Surgery, The University of Tokyo Hospital, Hongo 7-3-1, Bunkyo-ku, Tokyo 113-8655, Japan; ^3^Department of Pathology, Cancer Institute Hospital, Ariake 3-8-31, Koutou-ku, Tokyo 135-8550, Japan

## Abstract

*Background*. Soft tissue sarcoma with a hemorrhagic component that cannot be easily diagnosed by needle biopsy is defined here as soft tissue sarcoma with telangiectatic changes (STST).* Methods*. We retrospectively reviewed clinicopathological data of STST from 14 out of 784 patients (prevalence: 1.8%) with soft tissue sarcoma.* Results*. Tumors were found mostly in the lower leg. Histological diagnoses were undifferentiated pleomorphic sarcoma (*n* = 5), synovial sarcoma (*n* = 5), epithelioid sarcoma (*n* = 2), and malignant peripheral nerve sheath tumor and fibrosarcoma (*n* = 1). No history of trauma to the tumor site was recorded in any patient. Needle aspiration transiently reduced the tumor volume, but subsequent recovery of tumor size was observed in all cases. Out of 14 patients, 9 presented with a painful mass. MRI characteristics included intratumoral nodules (64.3%). The local recurrence rate was 14.3%, and the 2-year event-free survival rate was poorer (50%) than that of most sarcomas.* Conclusions*. STST is unique in its clinicopathological presentation. Painful hematomas without a trauma history, intratumoral nodules within a large hemorrhagic component, and subsequent recovery of tumor size after aspiration are indicative of the presence of STST.

## 1. Introduction

Needle biopsies are useful for histological diagnosis, since most soft tissue sarcomas are solid. However, small segments of soft tissue sarcoma cannot be diagnosed by needle biopsy because the tumor contains a large, fluid-filled hemorrhagic component. This phenomenon is generally not well appreciated, resulting in delayed diagnosis and poor prognosis [1]. We defined this tumor as a soft tissue sarcoma with telangiectatic changes (STST). Fourteen patients were examined for their unique clinicopathological features, imaging characteristics, diagnostic procedures, and long-term outcomes. The aims of this study are to raise awareness of a rare but distinct clinical form of STST and to assist clinicians in distinguishing between STST and other similar pathologies (hematomas, benign tumors, etc.).

## 2. Patients and Methods

We retrospectively reviewed 764 cases of soft tissue sarcoma treated in our hospital between 1984 and 2006. Among these patients, 14 (1.8%) developed soft tissue sarcoma of the extremities, which presented with a significant hemorrhagic mass on MRI prior to the initiation of chemotherapy.

The mean age of these patients was 35.9 years (range: 18–66 years). The male to female ratio was 1.8 : 1. The mean follow-up period was 6 years (range: 1–19 years) after the diagnosis of STST.

Of these 14 patients, 7 developed lung metastases, 2 developed lymph node metastases, and 1 developed subcutaneous metastases. Seven patients had died of exacerbation of lung metastases. Information was reviewed from medical records detailing demographic characteristics, clinical presentation of the primary tumor, imaging characteristics, local recurrence of the primary tumor site, treatment procedure of the primary site and any lung metastasis, the diagnostic procedure, and oncological outcome. The clinical features of STST were subsequently analyzed.

## 3. Results

### 3.1. Clinical and Pathologic Characteristics

No history of trauma to the tumor site was recorded in any patient. The primary complaints were swelling (14 cases), pain (9 cases), and local inflammation (7 cases). Most tumors were located in the thigh (7 cases), followed by the lower thigh (3 cases), buttocks (2 cases), and knee and neck (1 case each).

All cases were categorized as high-grade sarcomas, with histological diagnoses of undifferentiated pleomorphic sarcoma (UPS; 5 cases), synovial sarcoma (5 cases), epithelioid sarcoma (2 cases), and malignant peripheral nerve sheath tumor (MPNST) and fibrosarcoma (1 case each) (Table 1). Tumor size was >5 cm in 12 cases and <5 cm in 2 cases.

### 3.2. Imaging Characteristics

**Figure 1:**
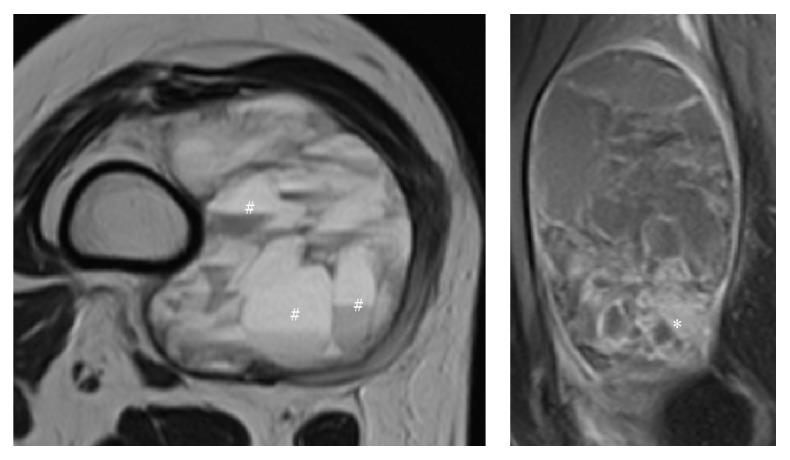
MRI characteristics of soft tissue sarcoma with telangiectatic changes (STST) images showed typical MRI features of STST: (#) fluid-fluid levels, (*∗*) tumor nodules.

MRI was performed in 10 out of 14 patients (71%). All tumors were observed to contain blood in over 70% of the tumor area. Tumor nodules on the side of the blood-filled mass were observed in all cases (Figure 1, *∗*), although the size of tumor nodules varied in each case. Four out of 10 cases contained fluid-fluid levels (Figure 1, #).

### 3.3. Diagnostic Procedure

Fine-needle aspiration was performed in 5 patients, resulting in a diagnosis of malignancy in only 1 patient. Needle aspiration temporarily reduces tumor volume, but subsequent recovery of the tumor size was observed in all cases. Core needle biopsies were performed in 6 patients; cytological diagnosis is a sensitivity of 57.1%, but histological diagnosis is a sensitivity of 28.6%. Open biopsies were performed in 10 patients because an accurate diagnosis could not be determined by needle biopsy in 8 cases, and small size precluded needle biopsy in the remaining 2 cases. Incisional and excisional biopsies were performed in 6 and 4 patients, respectively, with a sensitivity of 100% (Table 2). The mean interval between the first visit and pathologic diagnosis was 4 months (Table 3). Repeated aspiration of the tumor after recovery of the tumor size resulted in delayed diagnosis of STST of up to 36 months, because the tumors present as fluctuating masses that may be confused with a hematoma.

### 3.4. Treatment and Prognosis

**Figure 2:**
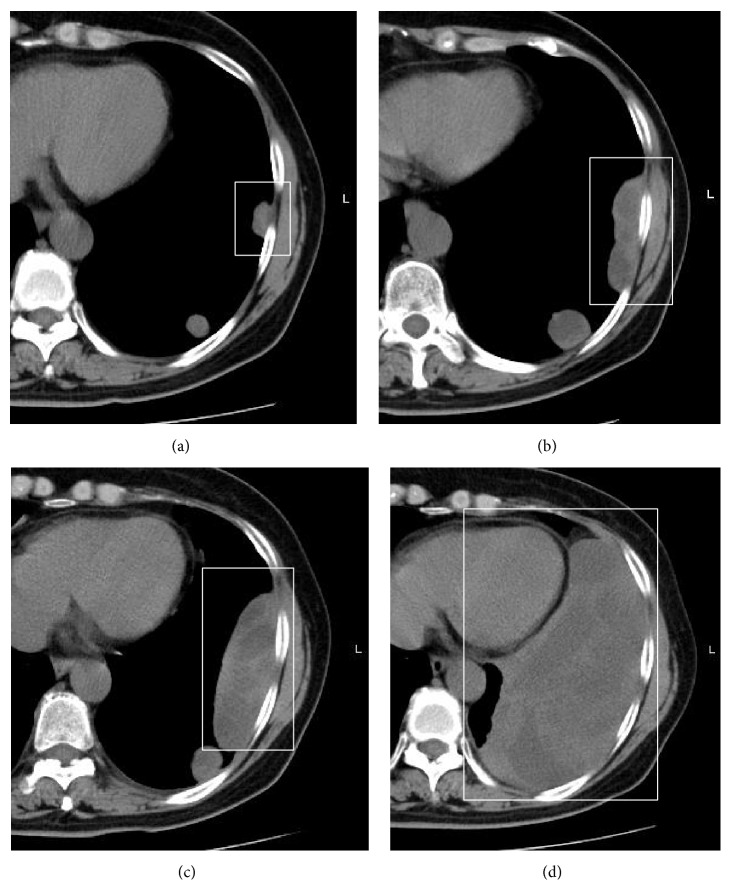
Lung metastases also presented telangiectatic change and resulted in rapid deterioration. (a) First detection of lung metastases. (b) Two months after first detection. (c) Three months after first detection. (d) Four months after first detection.

Wide resection of tumors with limb-sparing surgery was performed in 11 cases, and amputation was performed in 3 cases, resulting in wide-margin tumor resection in all cases. Local recurrence was observed in 2 cases (14%) and was not related to biopsy procedures and surgery (Table 2). The rate of metastasis was 50%, involving lungs (7 cases), lymph nodes (2 cases), and subcutaneous tissue (1 case). Out of the 7 cases of lung metastasis, an acute increase of lung nodules was observed because of telangiectatic changes in 3 cases (Figure 2). Chemotherapy was performed in all 7 cases of lung metastases postoperatively, but all 7 patients died of complications resulting from lung metastases. The 2-year event-free survival (EFS) rate was 50%.

## 4. Discussion

Weiss and Enzinger reported on this rare subset of tumors in their 1978 review of 200 cases of MFH [2]. The authors maintained that 5% of MFHs undergo such extreme hemorrhaging that they present clinically as fluctuating masses, which may be confused with hematoma. This study revealed a rare but distinct clinical form of STST. The prevalence of STST was reported to be in the range of 2.9–3.2% [3, 4]. In our study, the rate was relatively low, at 1.8%, as we analyzed only a subset of soft tissue sarcomas (i.e., those that had been difficult to diagnose by needle biopsies because of a hemorrhagic component). Telangiectatic changes occur in a variety of high-grade soft tissue sarcomas, such as MFH, primitive neuroectodermal tumors (PNET) synovial sarcoma, leiomyosarcoma, myxofibrosarcoma, and epithelioid sarcoma. However, intermediate grade sarcoma (e.g., angiomatoid fibrous histiocytoma) and benign tumors (e.g., hemangiomas and schwannomas) could present with a hemorrhagic change [5]. The anatomic distribution defining the presentation of STST correlates well with the sites for soft tissue sarcomas observed in our study, as reported previously [3].

Most high-grade STSTs often present as a painless, gradually enlarging mass [6]. In our study, 9 out of 14 patients (64%) complained of a painful mass but had no prior trauma history. Sternheim et al. reported a higher rate of 80% [3]. The reason of pain from the mass may be caused by intratumoral bleeding and the resulting rapid stretching of surrounding tissues. The lesion is often initially misdiagnosed as a deep intramuscular hematoma for the following reasons: (i) swelling is rapid; (ii) imaging displays a fluid-filled mass; and (iii) hematomas are more common than sarcomas [3].

In our study, repeated puncture of the masses misdiagnosed as hematomas led to a delay of STST diagnosis and treatment. Therefore, subsequent recovery of tumor size after aspiration is an important clue to the presence of STST [1, 7, 8].

In imaging, chronically expanding hematomas have been shown to have large central collections of heterogeneous signal material in both T1- and T2-weighted MRI series [9]. Unlike STSTs, hematomas are usually surrounded by a thick pseudocapsule of material (with a very low intensity T2 signal) composed of fibrous tissue, hemosiderin deposits, and iron-laden macrophages [10]. Therefore, the presence of the nodule enhanced in the tumor is often used to distinguish between hematomas and hemorrhagic neoplasms on MRI and CT scans. In our study, tumor nodules were observed in all cases. However, chronically expanding hematomas with intratumoral nodules have been reported in some instances [10]; therefore, the presence of a nodule does not definitively indicate the existence of a malignancy. On the other hand, a hemorrhagic mass without a nodule could be observed in a hematoma or a benign tumor but not in STST. The presence of fluid-fluid levels was observed in 40% of cases (4 out of 10). However, fluid-fluid levels can be observed in several types of benign tumor, such as hemangiomas and schwannomas; thus, this result did not help in differentiating benign from malignant neoplasms [5].

It is clinically and radiologically impossible to differentiate chronic hematomas from sarcomas; therefore, biopsies must be performed. Since STSTs tend to be misdiagnosed as hematomas as a result of imaging results, tumor aspiration is initially performed in most cases. In this study, fine-needle aspiration resulted in a diagnosis of malignancy in only 1 out of 5 patients. Another study reported a similar diagnostic rate of aspiration-biopsy cytology, that is, 1 out of 6 patients [1]. Fluid analysis showed the presence of numerous erythrocytes, a moderate presence of inflammatory cells (including lymphocytes, macrophages cells, and unaltered neutrophils) like those observed in hematomas. Even when no tumor or atypical cells are observed, reductions in mass size after aspiration followed by rapid mass recovery should be considered a clue to the possible presence of STST. In our study, repetitive aspiration-biopsy cytology alone would have led to the correct diagnosis of STST at the outset. Core needle biopsies were performed in 6 patients, demonstrating that cytological diagnosis was 57.1% sensitivity and histological diagnosis was 28.6% sensitivity. A past study reported a sensitivity of 100% in their subject population [3]. The low rate of diagnosis by core needle biopsy observed in our study was caused by an inability to target the small intratumoral nodule. By contrast, open biopsies were performed with 100% diagnostic sensitivity in our study. We performed excisional biopsies of tumors whose nodules were very small and thus not easily accessible. If internal patchy gadolinium enhancement is observed in the mass, a chronically expanding hematoma remains a possible diagnosis, but immediate aspiration, targeted biopsy, or surgical excision would be necessary to exclude the diagnosis of a hemorrhagic sarcoma because of overlapping imaging characteristics [10].

Sternheim et al. reported a relatively high local recurrence rate of 30% [3], substantially higher than the 14% rate (2 out of 14 cases) observed in our study (which is the same rate as overall soft tissue sarcomas); this is because a wide surgical margin was achieved in our cases. This evidence indicates that STST is not the manifestation of local aggressive behavior. In this study, the overall survival rate was 50%—a poor prognosis—confirming previous reports [3]. In our study, metastatic lung lesions caused intratumoral hemorrhaging and acute increases in tumor size, which, in addition to delayed diagnoses, resulted in a poorer patient prognosis and a relatively more rapid deterioration than those observed in prior reports.

## 5. Conclusion

STST is a rare and unique clinicopathological entity. STSTs present with characteristic MR imaging patterns, but precise differentiation from benign neoplasms is challenging. However, reduction in size after aspiration and subsequent rapid recovery of tumor size are suggestive to the clinician of the possible presence of STST, and cytological and pathological analyses should be performed through repeated aspiration and needle biopsies. In the instance that no malignancy is observed by needle biopsy, incisional and excisional biopsy should be performed to precisely diagnose STST. STSTs present with aggressive biological behavior and hemorrhagic changes, even at the metastatic lesions; therefore, prompt excision of the metastatic lesions could improve overall outcomes.

## Figures and Tables

**Table 1:** Clinicopathological characteristics of 14 patients with soft tissue sarcoma with telangiectatic changes (STST).

Sex	
Male	9
Female	5
Trauma	
+	0
−	14
Clinical presentation	
Swell	14
Pain	9
Local inflammation	7
Anatomical location	
Thigh	7
Lower thigh	3
Buttock	2
Knee	1
Neck	1
Histological diagnosis	
UPS	5
Synovial sarcoma	5
Epithelioid sarcoma	2
MPNST	1
Fibrosarcoma	1

UPS: undifferentiated pleomorphic sarcoma.

MPNST: malignant peripheral nerve sheath tumor.

**Table 2:** Relationship among biopsy strategy, surgery, and local recurrence in 14 patients.

Case number	Needle biopsy			Open biopsy		Surgery	Local recurrence
	FNA	CNB		Incisional biopsy	Excisional biopsy		
		Cytology	Histology				
1	Negative	—	—	+	−	Amputation	−
2	Negative	—	—	+	−	Amputation	−
3	Negative	—	—	−	+	Limb-sparing surgery	−
4	—	—	—	+	−	Limb-sparing surgery	−
5	—	Negative	Neurogenic tumor	−	+	Limb-sparing surgery	−
6	—	—	—	−	−	Limb-sparing surgery	−
7	—	Positive	Spindle cell tumor	+	−	Amputation	+
8	Positive	—	—	−	−	Limb-sparing surgery	−
9	Negative	Negative	Fat tissue	−	+	Limb-sparing surgery	−
10	—	Positive	Spindle cell tumor	+	−	Limb-sparing surgery	−
11	—	Negative	Hemangioma	−	+	Limb-sparing surgery	−
12	—	Positive	UPS	−	−	Limb-sparing surgery	−
13	—	—	—	+	−	Limb-sparing surgery	−
14	—	Positive	UPS	−	−	Limb-sparing surgery	+

FNA: fine-needle aspiration.

CNB: core needle biopsy.

UPS: undifferentiated pleomorphic sarcoma.

**Table 3:** Interval between first visit and diagnosis in 14 patients.

Interval (month)	
0	3
1	7
3	1
6	1
36	1
Unknown	1
